# ‘It Would've Been Nice to Know About Allied Health Earlier’: Insights From People With Parkinson's Disease

**DOI:** 10.1111/hex.70391

**Published:** 2025-08-21

**Authors:** Cassandra M. Wong, Sarah M. Dennis, Natalie E. Allen, Serene S. Paul

**Affiliations:** ^1^ Sydney School of Health Sciences, Faculty of Medicine and Health The University of Sydney Sydney Australia; ^2^ South Western Sydney Local Health District Liverpool Australia; ^3^ Ingham Institute for Applied Medical Research Liverpool Australia

**Keywords:** allied health, barriers to access, multidisciplinary care, Parkinson's disease, qualitative research, referral patterns

## Abstract

**Introduction:**

Allied health interventions can improve impairments and quality of life for people with Parkinson's disease (PwPD). However, allied health services are underutilised, and PwPD encounter barriers when accessing allied health. This study examined the allied health referral patterns of PwPD in New South Wales, Australia, from their perspective.

**Methods:**

Community‐dwelling PwPD and their care‐partners (CPs) were recruited. Participants completed a questionnaire, two‐stage semi‐structured interviews and a structured retrospective chart to track their PD journey.

**Results:**

Eighteen PwPD and five CPs participated, including six from culturally and linguistically diverse (CALD) backgrounds, two of whom required an interpreter. The Levesque model of healthcare access was utilised to describe this study's themes. The *approachability* and *appropriateness* of allied health services varied, as did participants' *ability to perceive* the need for services. CALD participants' fluency in English further impacted their perceptions, and they often found traditional medicine more *acceptable*. Health service *availability* was limited, particularly when accessing multidisciplinary care and in regional areas. Participants who lived alone or did not drive had limited *ability to reach* services. A lack of *affordable* services and limited *ability to pay* contributed to difficulties accessing allied health interventions; this could be somewhat relieved by funding packages. The *ability to seek* and *engage* in healthcare was present in all participants.

**Conclusions:**

PwPD recognise the need for allied health but experience barriers when accessing care, resulting in them not receiving the recommended early, regular and ongoing allied healthcare. Funding arrangements should be reviewed to enable this.

**Patient or Public Contribution:**

People with Parkinson's disease and their care‐partners generated the findings of this study through their interviews and retrospective charts. They provided feedback on results via member checking of their transcripts.

**Clinical Trial Registration:**

Not applicable.

## 1 Introduction

Globally, the prevalence of Parkinson's disease (PD) is increasing, with 11.8 million people living with PD in 2021 [1, 2]. PD represents a significant health system cost burden, with $52 billion spent annually in the United States [1]. People with PD (PwPD) experience motor and non‐motor symptoms (e.g., bradykinesia, cognitive impairment and anxiety) which significantly impact their quality of life (QoL) [3, 4, 5, 6, 7]. Multidisciplinary team (MDT)‐based care is important for managing PD [1].

Allied health interventions from physiotherapy (PT), occupational therapy (OT), speech pathology (SP) [6, 8, 9, 10, 11], social work, psychology and dietetics [12, 13, 14] are beneficial for PwPD [9]. They improve mobility, activities of daily living (ADLs), speech, swallowing, mood and QoL and reduce falls [3, 4, 15, 16, 17, 18, 19, 20, 21, 22, 23]. These interventions are particularly effective when they are coordinated by a team [12, 24] and delivered early in the disease [25, 26]. Despite recommendations that MDT care be delivered proactively and regularly [25, 26] (6‐ to 12‐monthly) to optimise function and QoL [3, 4, 15, 16, 17, 18, 19, 20, 21, 22, 23], allied health services are underutilised globally [10, 11, 25, 27, 28, 29]. Barriers to access for PwPD have been found at both person and system levels, including cost, autonomy, health literacy and beliefs, communication, coordination of care, and a disparity or unavailability of relevant services [30, 31].

Australian healthcare comprises both public (Medicare subsidised) and private services, similar to Canada, France and Belgium [32]. Community‐based allied health services are largely accessed via private health insurance or fee‐for‐service [33, 34]. Allied health can also be accessed through public funding schemes such as the National Disability Insurance Scheme (NDIS) [35] for those aged < 65 years, or My Aged Care [36] for those ≥ 65 years, but these schemes have strict eligibility criteria. A limited number of Medicare‐subsidised allied health sessions can be accessed through a general practitioner (GP) referral [37, 38].

Some research has investigated the lived experience of PwPD, including healthcare [39], past/present experiences and future expectations [40], but they have not examined PwPD's thoughts on management or reasons for seeing allied health. Since little is known about the timing of and triggers for allied health referral, this study sought primarily to determine allied health access, barriers and facilitators, and secondarily to determine early‐stage management and triggers for allied health referral.

## 2 Materials and Methods

This qualitative study involved two‐stage in‐depth semi‐structured interviews and completion of a structured retrospective chart (‘PD diary’). These were chosen to facilitate the participant's recall of events, particularly for those with cognitive impairment or long‐standing disease. Data were analysed openly and inductively using thematic analysis [41] following a constructivist interpretivist position [42, 43], which focused on how participants constructed their own meanings within their context from their individual experiences with PD. Inductive thematic analysis was chosen so that patterns could be identified without the use of predetermined ideas or categories, and data presented in rich detail.

This study was approved by the University of Sydney Human Research Ethics Committee (2022/892) and is reported according to the Consolidated Criteria for Reporting Qualitative Research (COREQ) checklist [44].

### 2.1 Participants

Community‐dwelling adults with a diagnosis of idiopathic PD, across the Hoehn and Yahr [45] spectrum of disease stages, residing in New South Wales (NSW), Australia, were included. PwPD who were unable to participate independently could take part if they had a care‐partner (CP; e.g., family member or close friend) to assist. When needed, interpreters were provided for culturally and linguistically diverse (CALD) participants (i.e., people who were born overseas, whose parent was born overseas or speak a variety of languages [46]). Recruitment occurred between January 2023 and August 2024 (Appendix A).

### 2.2 Data Collection

All participants provided informed consent. Data collected via questionnaire included demographics, physical activity levels [47], private health insurance cover and a health literacy screening question [48] (Appendix A). PD‐related questions included fall history, year of diagnosis, disease severity [45], medication, and non‐motor and motor experiences of daily living [49]. Questionnaire data were used to guide purposive sampling to ensure diversity in socio‐economic status [50], geographical location [51], health insurance status, gender, disease stage and CALD representation.

Participants completed two semi‐structured interviews and a structured retrospective chart (Appendix B). The interview guide (Appendix C) and chart were piloted on an experienced PD researcher without PD. Interview 1 provided an overview of the participant's PD journey, their diagnosis and health professional involvement. Participants were introduced to the chart and asked to complete it before Interview 2. The chart, adapted from the Life Chart Manual [52], where an individual retrospectively charts their psychiatric history, was used to retrospectively document each participant's PD progression and care‐seeking triggers. Participants recorded changes in symptoms, medications, falls and allied health involvement from diagnosis to present. At Interview 2, the chart was reviewed in depth to enhance insight into the participant's PD journey, including triggers for and factors influencing allied health access. CALD participants were provided with translated charts as needed (Appendix A).

Interviews were conducted at the University of Sydney (*n* = 1), the participant's home (*n* = 16), their respite facility (*n* = 1), or via Zoom (*n* = 19), between March 2023 and September 2024. Interviews were conducted by a female researcher (C.W.), a physiotherapist experienced in treating PwPD with qualitative training who had no prior interaction with participants. Table 1 provides further detail on the processes utilised to ensure qualitative rigour [53, 54, 55].

**Table 1:** Dimensions of rigour.

Dimension	Strategies
Credibility	Prolonged engagement The interviewer (C.W.) called participants to recruit them or called them after they had provided their contact details to express interest in the study. During this phone call, the interviewer answered questions and introduced them to study materials and procedures, which were reiterated at the start of the first interview. Given two‐stage interviews were conducted, the interviewer had time to build rapport with participants and to explore perspectives they had expressed in the first interview in‐depth in the second interview. Interviewing process and techniques The interviewer undertook training in qualitative methods and interview techniques before commencing this study. The interview and PD diary were pilot tested on an experienced PD researcher without PD and modifications were made following the feedback provided. The interview prompts were flexible and could be utilised at different time points during the interviews to enable participants to expand on their thoughts. Interview prompts were discussed during team debriefs and adjusted as needed. Establishing investigators' authority Three researchers were experienced neurological clinicians (C.W., N.A. and S.P.), with two of these being PD expert researchers (N.A. and S.P.). All researchers had qualitative expertise and were familiar with interviewing techniques and data analysis utilising NVivo software (C.W., N.A., S.D. and S.P.). One team member was a health services researcher (S.D.). All participants were aware that the interviewer was a physiotherapist completing her PhD. Collection of referential adequacy materials Field notes and reflexive memos were kept for every participant. A coding journal was kept and updated as coding of transcripts progressed. These materials were referred to and discussed during team meetings.
Dependability	Study protocol description The protocol for this study was developed following a review of the literature about allied health access and Parkinson's disease and built on previous work conducted by research team members. All authors contributed to the development of the study protocol, which was submitted for and received ethics approval. Modifications made to the protocol were documented and tracked. Establishing an audit trail Data collection dates and times were recorded and stored in a secure central file. All transcripts were checked for accuracy by comparing them against their original audio file. Coding progress was regularly discussed by the team, with all members agreeing on modifications to be made. The coding journal was updated to record decisions made in the meetings and to reflect the changes made to codes as a result.
Confirmability	Reflexivity The interviewer was mindful of the potential interviewer‐interviewee power imbalance and sought to ensure participants were comfortable before commencing the interview and utilised the opening questions to continue rapport building before progressing to more in‐depth questions. All participants were aware that the interviewer was a physiotherapist completing her PhD, which did lead to conversations about physiotherapy. The interviewer is aware that this knowledge may have contributed to participants portraying physiotherapy in a positive light. The interviewer ensured participants also discussed their knowledge or usage of other allied health disciplines to ensure a holistic view of healthcare access could be gained. The researchers were aware that their prior experiences and motivations may have influenced the decisions made throughout the project. All researchers brought different experiences and perspectives to the project. Differences in perspective and potential personal biases were discussed during team meetings. The interviewer kept reflexive memos throughout the interview and analysis process that were referred to during team discussions. Triangulation Data source triangulation occurred by comparing participants' interviews and structured retrospective charts to other participants of different disease stage, socioeconomic status, geographic location, gender or CALD group to determine what experiences were similar and what differed. Investigators' triangulation was achieved through research team meetings where agreements about coding decisions were reached.
Transferability	Purposive sampling Purposive sampling was utilised to ensure a mix of gender, disease stage, socio‐economic status, geographic location and CALD groups, to ensure diverse perspectives of PD and healthcare access were gained. Data saturation Theoretical saturation was discussed regularly at team meetings. Saturation was determined to be achieved when no new codes and concepts were being identified. Theoretical saturation was achieved first for English‐speaking participants, and then for CALD participants.

All interviews were audio recorded, with field notes made during and immediately following the interview. The interviewer (C.W.) debriefed with another researcher (S.P. or S.D.) after every interview to determine further sampling, modify prompts and review data saturation. Recordings were transcribed verbatim by researchers (C.W. or S.P.) or a professional transcription service. Transcripts were returned for member checking to consenting participants; one returned minor edits.

### 2.3 Data Analysis

Chart data were utilised to create timelines to track PD journeys, identify symptom progression and triggers for seeking care. The researcher (C.W.) familiarised herself with the data by listening to the interviews and reading the transcripts. Data was coded line by line and analysed thematically [41] using NVivo software v14 (QSR International Pty Ltd, 2023).

Researchers met regularly to refine the coding framework. As analysis progressed, many initial codes relating to the primary aim were found to align with the Levesque model of healthcare access [56], which categorises healthcare accessibility into five supply‐side and five demand‐side determinants (Table 2). The coding tree was therefore revised to incorporate this model.

**Table 2:** Levesque model of healthcare access [56].

Supply‐side determinants		Demand‐side determinants	
Approachability	Whether a person can identify that a service exists, whether it can be reached and if it can have an impact.	Ability to perceive	A person's ability to perceive the need for healthcare is based on their health literacy, knowledge and beliefs.
Acceptability	How appropriate it is to seek care as determined by a person's social and cultural factors.	Ability to seek	Ability to seek healthcare is influenced by a person's autonomy and capacity, as well as their knowledge of healthcare and rights.
Availability and accommodation	Whether a health service exists, and if it has the capacity to deliver services promptly.	Ability to reach	The ability to reach healthcare services is dependent on a person's mobility, access to transportation, healthcare knowledge and flexibility to take time away from work to attend appointments.
Affordability	The cost of the service, both in time and money.	Ability to pay	Ability to pay for healthcare refers to the capacity of a person to generate economic resources that they can use to pay for health services.
Appropriateness	How well a service fits a person's needs, including treatment quality and timeliness.	Ability to engage	Ability to engage in healthcare refers to how a person participates in treatment and decision making, impacted by their capacity, motivation, health literacy and self‐efficacy.

After interviewing eight English‐speaking participants with private health insurance living in metropolitan or inner regional areas, the researchers determined that data saturation for this group was imminent. Purposive sampling then focused on participants without health insurance or living in outer regional areas. After four additional interviews, data saturation was reached for English‐speaking participants. Purposive sampling of CALD participants was undertaken, with data saturation after six interviews.

## 3 Results

Eighteen PwPD and five CPs (all spouses, who participated as a dyad with the PwPD) participated (Table 3 and Appendix D). One CALD participant and their CP withdrew after Interview 1, but gave permission for their data to be included. Two participants and their CPs were interviewed with Cantonese‐speaking interpreters. One of these completed four interviews over 11 weeks to accommodate the time needed for interpretation and the participant's limited capacity for long sessions. Sixteen participants completed the two‐stage interviews over 1–5 weeks. Total interview times ranged from 65 to 172 min. Two PwPD (including one CP) had not completed their chart by Interview 2, so the interviewer facilitated its completion during the session.

**Table 3:** Characteristics of the study participants. Data reported as *n* (%) or median (range).

Characteristic	Participants *n* = 18
Sex, male, *n* (%)	12 (66.7)
Age at time of study	70.5 (42–84)
Estimated age at diagnosis	58 (38–80)
Years since diagnosis	9 (3–32)
Hoehn and Yahr stage, *n* (%)	
1	6 (33.3)
2	3 (16.7)
3	3 (1.67)
4	6 (33.3)
5	0
MDS‐Unified Parkinson's Disease Rating Scale (MDS‐UPDRS)	
Part 1A (0–20)	5 (0–14)
Part 1B (0–28)	10 (1–20)
Part 2 (0–52)	17 (1–47)
Residential location^a^, *n* (%)	
Major cities	14 (77.8)
Inner regional	3 (16.7)
Outer regional	1 (5.6)
Index of Relative Socio‐economic Advantage (ISRAD)^b^	
1 (most disadvantaged)	1 (5.6)
2	0
3	3 (16.7)
4	3 (16.7)
5 (most advantaged)	11 (61.1)
Languages spoken, *n* (%)	
Number who spoke more than 1 language	6 (33.3)
English	16 (88.9)
Cantonese^c^	3 (16.7)
French	1 (5.6)
Japanese	1 (5.6)
Mandarin	1 (5.6)
Spanish	1 (5.6)
Swedish	1 (5.6)
Teochew	1 (5.6)
Highest education level, *n* (%)	
Some high school	2 (11.1)
Completed high school	2 (11.1)
Completed TAFE^d^ or trade certificate	4 (22.2)
Completed university or college advanced education	10 (55.6)
Confidence completing medical forms, *n* (%)	
Not at all	2 (11.1)
A little bit	0
Somewhat	2 (11.1)
Quite a bit	3 (16.7)
Extremely	11 (61.1)
Living situation, *n* (%)	
By themselves	2 (11.1)
With spouse or partner	15 (83.3)
With other family	1 (5.6)
Working situation, *n* (%)	
Working full time	2 (11.1)
Working part time	1 (5.6)
Retired—pensioner	7 (38.9)
Retired—self‐funded	7 (38.9)^e^
Unemployed	1 (5.6)^e^
Health insurance, *n* (%)	
Private hospital coverage only	3 (16.7)
Private hospital coverage, but unsure about ancillary coverage	2 (11.1)
Private hospital and ancillary coverage	9 (50.0)
None	3 (16.7)
Department of Veterans Affairs (DVA) gold^f^	1 (5.6)
Other funding, *n* (%)	
My Aged Care	5 (27.8)
National Disability Insurance Scheme (NDIS)	6 (33.3)
Number of fallers in last 12 months, *n* (%)	10 (55.6)
Number of falls in last 12 months	2 (0–52)
Parkinson's disease medications^g^, *n* (%)	
Anticholinergic agents	0
Dopaminergic agents	
Dopa and dopa derivatives	24 (51.1)
Adamantane derivatives	4 (8.5)
Dopamine agonists	11 (23.4)
MAO‐B inhibitors	7 (14.9)
Other dopa agents	1 (2.1)
Incidental and Planned Exercise Questionnaire (Version W) (IPEQ‐W) Total Activity Score	24.1 (1.8–51.1)

^a^ Classified using the Australian Statistical Geography Standard Remoteness Areas Structure 2021 [51].

^b^ Classified according to Socio‐Economic Indexes for Areas (SEIFA) 2021 [50].

^c^ Two participants were interviewed with Cantonese interpreters.

^d^ Technical and Further Education (TAFE).

^e^ *n* = 1 indicated they also completed home duties.

^f^ Department of Veterans Affairs covers those who serve/served in the defence force and conditionally their spouses or families.

^g^ Multiple participants took more than one medication.

### 3.1 Barriers and Facilitators to Allied Health Referral

The Levesque model [56] was utilised to describe the barriers and facilitators PwPD encountered when seeking and receiving allied health. Supply‐side determinants relate to features of systems and organisations that influence access to healthcare, while demand‐side determinants reflect the corresponding features of populations and the process of accessing healthcare. Each corresponding supply and demand determinant is described below. Additional quotes are presented in Table 4.

**Table 4:** Quotes.

Results section	Participant quote
***Barriers and facilitators to allied health referral***	
Ability to perceive	‘*[I researched] on the internet mostly I think. One of them [speech therapy program] I heard about. I've read about them with the Michael J Fox thing, and where else was I that heard? Oh, I think when Parkinson's NSW one thing they did had an article on it there. I think that's where I first heard about Punching for Parkos too…’* PwPD2
Acceptability and ability to seek	‘*He went back to China to seek medical treatment because he was told that this disease as far as western medicine's concerned it can only be like controlled but not cured. So, he went back to China with the hope that he could get better.’* CP16‐CALD ‘*if there was anybody that I did need to go and see for advice, the current—the health professionals I do know would put me straight onto them.’ PwPD11*
Availability and accommodation	‘*For the physio she [GP] was like “…we have like one that we recommend that's near the GP clinic…” [But I've found] there's a place that's right over the road from where I live that basically is a physiotherapist… I like the idea that it's nearby, so I can just duck over, sort of 5 min before the appointment…’* PwPD5 ‘*Even if I'm tired, I think, I can get to [new EP practice] and back… so I think it's working for me, even though I do think [previous practice] was the best, really… when I went before we lived in [suburb], which is just that bit closer… and parking isn't always good there. So, sometimes if you go there driving feeling really tired—whereas 10 min [to new EP practice], I can—it's not a problem.’* PwPD2 ‘*[Neurologist] recommended… that we get a speech therapist. Which is extremely, extremely hard unless you do it online. Which… well, I'm not comfortable with.’* CP3
Affordability and ability to pay	‘*The girl… said to me, have you applied for NDIS money? And I didn't know anything about it…. I was diagnosed at 58, so I was eligible…. I thought, you know it's good for covering because it does add up. Exercise physiologists especially are pretty expensive… at $166 a pop I wasn't going to be able to go all the time you know. So, after I got the funding, I started to go on a more regular basis.’* PwPD2 ‘*Well, for a period of time, he went for physio treatment two times a week. Those days, NDIS paid for it. Then later on, not enough funding. So we stopped. That period of time lasts for around three months.’* CP16‐CALD
Appropriateness and ability to engage	‘*The first one [SP], I think the Parkinson's Association recommended her… she was doing the LSVT programme…. And so I started with her. Then I think I looked up, the second one was local to me, and I think I looked her up on the internet maybe. I think I googled LSVT and just happened that she, she'd done that course so…’* PwPD7 ‘*I didn't request for those treatment but the doctor was very kind because I told him he got swallowing problem, so he arranged speech pathology to see my husband [in hospital]. He referred us to see the physiotherapist as well.’* CP19‐CALD ‘*I've just come to the stage in my life now I think I've really gotta do something about it, in that I can, I can't get rid of it, but I can delay it so…. And that there are a lot of resources out there that I didn't know about that I do know about now, and I'm sure there's more I'm going to learn going forward.’* PwPD4
***Early‐stage management and expectations***	
Education provided by a neurologist	‘*I mean, one of the resources was encouraging and I was encouraged to do exercise. [Neurologist recommended] An exercise place.’* PwPD1
Desire for more information	‘*They [doctors] should have some sort of package that you know, they could just give to you when you've been diagnosed, cause it's quite overwhelming really. Just from, you know, just from the medical side of things, let alone the, the activity side of things…. I just wasn't aware that this was around.’ PwPD4*
Changed perceptions	‘*I would like to say that if earlier we have so much information, then we may not need to go to China for the [deep brain stimulation] surgery…. I feel that the Chinese doctor—they do not have as much information. They do not know this disease too well.’* CP16‐CALD
Participants' advice to others	‘*Try and stay positive no matter what…. Keep moving, keep exercising.’* PwPD13 ‘*It's not a death sentence. And make the changes, the lifestyle changes early because the sooner you make them, the sooner you see the impact.’* PwPD5 ‘*I think he [Neurologist] advised to join a support group, which I did do, which was very helpful. The most important thing he said to me was—the two most important things are diet… and exercise… and that's what I've found since.’* PwPD11

#### 3.1.1 Approachability (Supply) and Ability to Perceive (Demand)

Approachability varied depending on how much information clinicians provided, participants' understanding of PD, and their connection to support networks.

Many were unaware of what health services they should be looking for.

‘I knew about allied health disciplines, generally, but I didn't know they had a specialised Parkinson's clinic…’

PwPD9

They relied on clinicians and peer networks for guidance.

‘It's only through… the OT and the Parkinson's nurse and then now the support group that, you know, all these things are out there that I possibly could have been doing for the last couple of years that I didn't know about them.’

PwPD4

Navigating funding systems like My Aged Care and the NDIS posed challenges, both at entry and during attempts to access package‐funded care.

‘I start the process of NDIS. I apply before but wasn't successful until I got Parkinson's NSW to help me apply. Lots of things that they ask for, I couldn't supply or didn't know how to supply to them. Until they help me out.’

PwPD15‐CALD

Ability to perceive the need for care was evident in all participants when they initially sought medical care, first seeing their GP and then a neurologist for diagnosis. However, health literacy and perceptions of allied health varied. Some actively researched PD, becoming aware of allied health services. While some engaged proactively with services, others only sought allied health intervention in response to symptoms. Perceived need differed across disciplines and between participants, with some choosing not to engage when they did not perceive the need.

‘No, I haven't [sought allied health]. I haven't because… it's not affecting my life that way at the moment, I'm pushing through. I mean, sure, there's different things like my speech does get funny sometimes, but you have good and bad days…’

PwPD6

CALD participants' English proficiency impacted their understanding of PD and management plans. Some were confident attending appointments without interpreters, while others were not. Few relied on family or friends for translation due to their lack of availability.

‘Well, no, because doctor is the Westerner, and in those days, I did not know that I could request interpreter… a lot of things that we didn't quite understand… I did not know what is Parkinson's and how bad, how severe this… sickness is…. Many years… maybe at least five to six years…. Then I noticed this is a serious problem.’

CP16‐CALD

CALD participants with supportive networks were more likely to be well‐connected to information and clinicians. Those without supportive networks were often less aware of available services and did not actively seek support.

‘I feel that we joined—the Parkinson disease patients' meeting [support group] is much better…. Well, the patients can talk to each other. They communicate with each other and share…. Sometimes, doctor would come to do some seminar and tell us how is the healthcare now, the latest development is like what. If you just stay home, no information…’

CP16‐CALD

#### 3.1.2 Acceptability (Supply) and Ability to Seek (Demand)

Acceptability was influenced by cultural background. Some participants, particularly those with CALD backgrounds, explored traditional or alternative therapies alongside Western medicine, especially when conventional treatments felt ineffective. Sometimes they preferred these to other allied health or Western interventions.

‘I found both his legs is weak for walking so actually I try acupuncture and massage…. Because my husband is under the cover of My Aged Care so I request the provider to arrange some acupuncture or massage for my husband. So, they found some for us and after the acupuncture and massage I've found there is a slightly improvement in my husband.’

CP19‐CALD

When seeking healthcare, participants valued having choice and control. They sought providers who met their needs and changed providers when dissatisfied. Similarly, participants wanted control in selecting allied health interventions, with some finding certain programmes too confronting in early disease.

‘when I first went to [Neurologist 1] who recommended I go to [hospital], they have a program, and it was kind of scary. Because I was still in very good shape at that time and they sort of said… “we'll take and record your base levels of… skill levels of things, but you'll need to bring a carer, and you won't be allowed to drive home…” They treated me almost immediately like an invalid. And you know which was really frightening. And when I talked to [Neurologist 2] about, you know, it's important that I go there. He said no.’

PwPD2

CALD participants often sought clinicians who could speak their language to facilitate communication.

‘The chiropractor is helpful because he speaks Chinese and so there's no problems in communicating.’

CP16‐CALD

Participants' knowledge about healthcare options varied. Proactive participants independently researched options and started allied health interventions. Others relied on clinicians for education and referrals. Some sought allied health support after receiving information from clinicians at support groups or through recommendations from friends or family.

‘I took my granddaughter out there because she had speech pathology… then I talked to them about Parkinson's and things. I went once to have a bit of an assessment and they said—basically, they said it wasn't too bad.’

PwPD2

#### 3.1.3 Availability and Accommodation (Supply) and Ability to Reach (Demand)

Distance and travel were commonly reported deterrents to accessing general healthcare and allied health. Many prioritised the convenience of seeing local therapists over travelling to higher‐quality services. However, for neurologists, they were often willing to travel.

‘…but it entailed traveling from where we are [regional area] to there [metropolitan, for the Neurologist]—that's 2 days round trip, accommodation, everything else—so a little bit of inconvenience…’

PwPD13

The availability of health services limited what interventions PwPD could receive, further limited by waitlist and follow‐up issues, particularly in regional areas. MDT access was particularly limited.

‘Yeah, it's [my speech is] getting worse now. I'm supposed to see a speech therapist, but they're hard to get, you can't find them anywhere.’

PwPD10

Even where services were available, sometimes eligibility criteria prevented access. This was particularly the case for MDT clinics, with one young‐onset participant describing how they were placed on a waitlist due to the mild severity of their PD.

‘she [GP] did refer me to a Parkinson's clinic at [hospital]. And they basically said, “look, we'll keep your details, but you are sort of like so low needs at the moment that we couldn't actually justify taking you on to our service.” …they've got all the services on site… I guess it's mixed feelings, both happy and unhappy. Sort of happy to know that I'm… not I guess debilitated enough to actually need that support, but at the same time it would be nice to have it.’

PwPD5

For some, telehealth was a barrier, whilst others found this useful.

‘Originally it was going to be face‐to‐face, but COVID intervened. Which worked out fine, anyway. I mean, basically I had my initial assessment done by Zoom and then I joined the… telehealth physio class, which was also by Zoom, and that all worked out fine.’

PwPD9

Ability to reach healthcare was influenced by living arrangements and transport access. Participants who could drive or had family to assist could access care. In contrast, those who did not drive or lived alone were limited to local services or relied on others for transport. Public transport was often unsuitable for those with more advanced PD.

‘I go to [neurologist at location]. My daughter takes me…. I think he said he want to see me once a year and then we do a telephone one every 3 months—no, FaceTime, so we can see each other… because it's a bit hard… nearly a whole day [for the appointment].’

PwPD17‐CALD

Some participants received home therapy, predominantly through NDIS and My Aged Care packages, which they found beneficial.

‘Well, I've got a home gym with a treadmill and a bike, so she [physio] gets me to use the treadmill and the bike.’

PwPD12

#### 3.1.4 Affordability (Supply) and Ability to Pay (Demand)

The high direct and indirect costs of allied health services limited ongoing access. PwPD discontinued services when costs were unaffordable or not subsidised by funding packages. Time costs, including appointments and travel, further impacted continued engagement.

‘I was given a series of vouchers for my 60th birthday, so I used those up and again it was…. It was just too much, expensive to do it, continue on… it's not that they haven't been good. It's just a financial side of things, you know, everything is so expensive.’

PwPD4

Younger participants and those with young‐onset PD were particularly concerned about the financial burden of accessing allied health.

‘a lot of it is the uncertainty about cost. I mean I've got kids, mortgage. Money is not incredibly tight, but it's still tight enough that it's a concern. And I know that if we needed to, like fund the $30 per visit for Physio that we'd be able to fund the money, but it's more what my family would be missing out on by me taking up that need.’

PwPD5

Approval of NDIS plans often led to increased access to allied health services. However, once funding was exhausted, participants often discontinued therapy.

‘In the current [NDIS] plan, I do [have enough funding]. In the first plan, I didn't…. They funded some, but not enough so I just didn't have therapy.’

PwPD12

In comparison to the NDIS, participants with My Aged Care received less funding for allied health, but did receive funding for assistance with ADLs and equipment. My Aged Care funded OTs most often.

‘And as a result of the aged care assessment that I had in 2021, it was recommended that I see a physio and I, again, just went back to that same physio and received more exercises. Also, an OT was recommended, and that's the one that I was particularly keen on, because they would examine the house and see what would be needed.’

PwPD13

Health insurance partially subsidised allied health, but gap payments and exclusions were burdens.

‘I do [have private health insurance]. That again only covers part of it…. I'd be out of pocket, I think it works out to be about $15 per half hour, and they [physio] would always want to book me in for an hour session. So, 30 bucks a week. Which doesn't sound like much until you've multiplied by the 52. You get the full amount for the year and then you go “hold on”.’

PwPD5

Some participants used GP mental health plans to subsidise psychology costs; however, these plans are capped at 10 visits.

‘after I was diagnosed…. Had a bit of a meltdown [saw a psychologist]… I think I got a, a mental health plan, but the first few I privately funded and then I got a mental health plan.’

PwPD7

#### 3.1.5 Appropriateness (Supply) and Ability to Engage (Demand)

Participants often did not know or understand the importance of seeking allied health clinicians with PD‐specific expertise. Once they perceived their need for care, they typically accessed local services, even when providers lacked relevant expertise (e.g., musculoskeletal PTs and paediatric SPs). Those who sought recommendations from neurologists, GPs or Parkinson's NSW were more likely to access PD‐specialised clinicians.

‘the fellow [physio] I saw didn't really know anything about Parkinson's, to be honest with you. He, I think he dealt more with sports injuries and stuff like that. So no, he didn't really give me any advice [about Parkinson's].’

PwPD6

**Figure 1:**
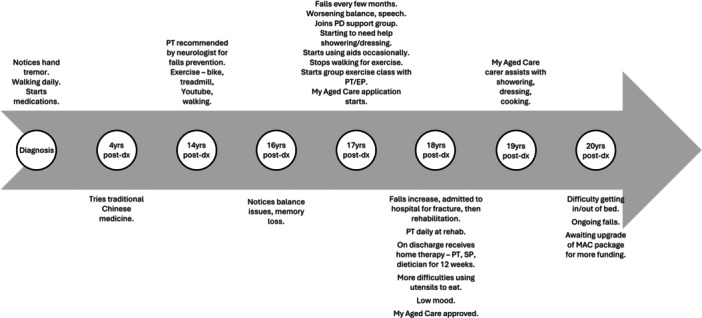
Example timeline of PwPD with long‐standing PD.

**Figure 2:**
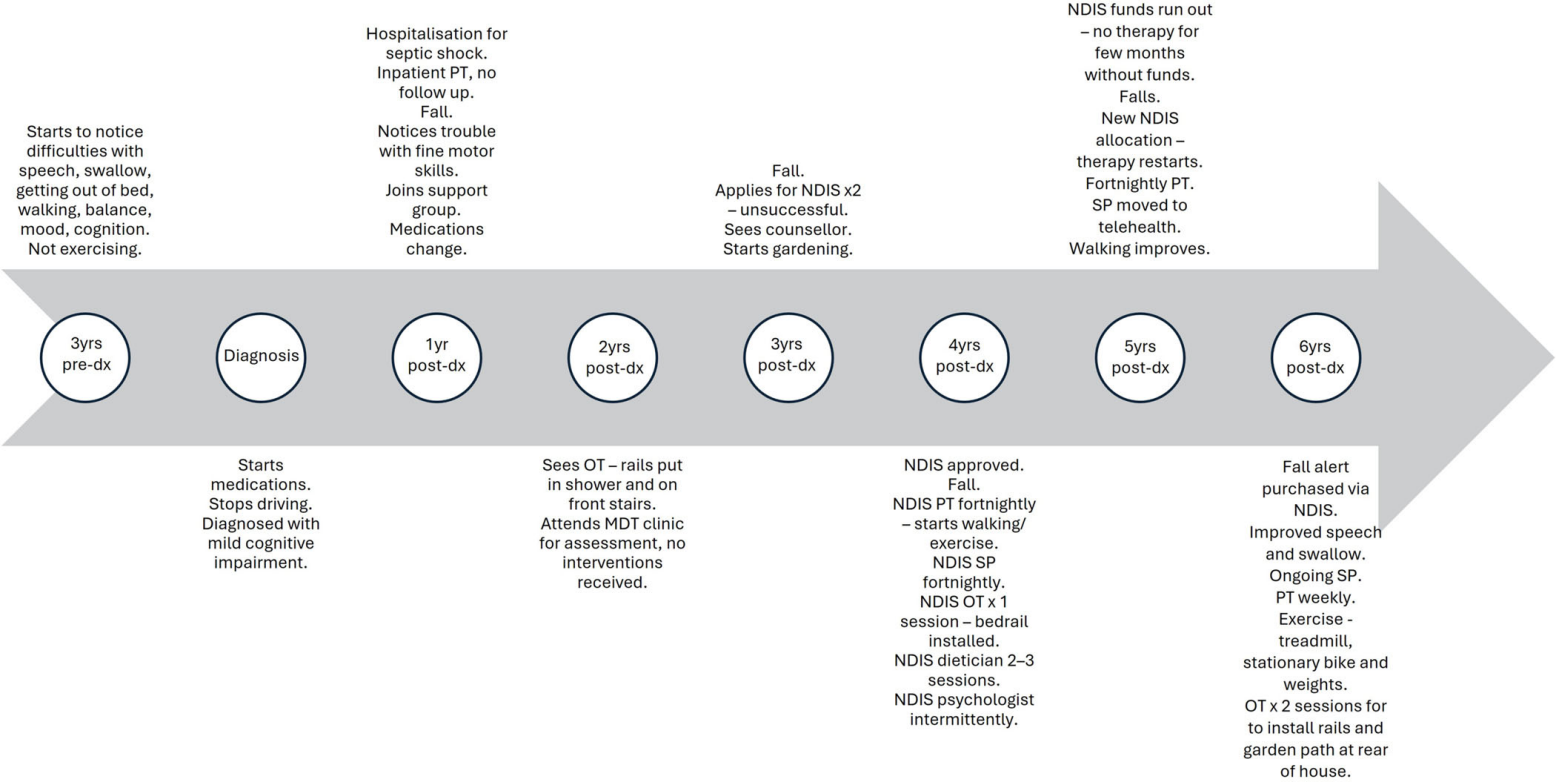
Example timeline of PwPD recently diagnosed.

Timeliness of allied health access varied, with participants accessing care both proactively and in response to worsening symptoms. PT/exercise physiology (EP) was most likely to be accessed proactively. Participants with a long‐standing PD diagnosis (Figure 1) were more likely to access allied health reactively than participants diagnosed more recently (Figure 2).

‘I went to a seminar when I was first diagnosed and one of the ladies… talked about how exercise and these big velocity exercises are the only thing you can really do that's positive to sort of help the progression. And so, I've always loved exercise…. So, I just, I just basically went up to [PT/EP practice] and saw them and then started from there.’

PwPD7

All participants were able to engage in healthcare decision‐making, though health literacy and self‐management skills varied. Some were more proactive than others, though attitudes could change over time. Participants described ceasing interventions when they deemed it no longer beneficial.

‘He did see one [psychologist] recommended by a friend but didn't continue because didn't feel it was helpful.’

CP16‐CALD

### 3.2 Early‐Stage Management and Expectations

Early management was predominantly medical. Education about PD and referral to allied health were variable, and satisfaction with the education provided was mixed (Table 4).

‘He [Neurologist] said it's a very slow progressing disease. And that not many people die of it…. And that he could help quite a bit with medications. And that was about it.’

PwPD2

In the early stages, participants had no clear expectations about management or their healthcare team. On reflection, they wished they had received more information earlier in their disease.

‘it would've been nice when I was first diagnosed to know that those [PT and multidisciplinary teams] were available, rather than having to go back through a GP and then the neurologist…’

PwPD9

PwPD and their CPs perceptions' on management changed over time, particularly if they were overwhelmed at diagnosis. Some who initially chose not to seek support or allied health interventions, or preferred traditional medicine, later sought allied health as their understanding changed.

‘I'm surprised I'm even saying this, but to join a support group. So therein lies the information that you need, and I was very, very against it because I said I don't want support…. But the Parkinson's… I've just found now, it's just opened my eyes to… there's so many other things there is to do and how important exercise is, which I didn't know.’

PwPD4

PwPD advised those newly diagnosed to stay positive, accept the diagnosis, seek information and support, and adopt a healthy lifestyle including exercise.

‘I think the important thing is that there is good support available, that you can access it, and that you should. Because the more you do the things that you can do early on, the more likely it is that you can delay the progression.’

PwPD9

### 3.3 Triggers for Allied Health Referrals and Disciplines

There was no consistent pattern to accessing allied health, and participants were not always referred by clinicians. Example timelines for a PwPD with long‐standing disease and recently diagnosed are presented in Figures 1 and 2. The allied health professionals seen most frequently were PTs, SPs, OTs, dieticians, EPs and psychologists, respectively. PwPD often had trouble differentiating between whether they had seen a PT or EP, particularly if they had attended a PD‐specific group exercise class. Four PwPD attended a multidisciplinary PD clinic at a public hospital for assessment, but not all received allied health interventions there. Only one PwPD reported seeing a counsellor, and one saw a PD nurse specialist (PDNS). While many had interacted with PDNSs at support groups, individual access was limited, and some expressed a desire for better availability.

Participants would often self‐seek allied health practitioners by locating local practices after researching PD, or following recommendations from family, friends or PD networks.

‘it was actually through the breast care nurse when I got breast cancer last year… the breast care nurse put me in touch with [PDNS]… I didn't even know she existed…. She rang me and said she'd like to come see me…’

PwPD4

Falling, deterioration in speech or swallowing, or hospitalisation did not consistently result in allied health referrals.

‘my medications were changed quite dramatically and I had a reaction to the changes and the increase…. I was in hospital for about 4 days, I think. [Seen by] just the general practitioners there [, no allied health].’

PwPD13

However, approval of a funding package frequently resulted in seeking allied health, often across multiple disciplines.

‘this was from just researching and once the NDIS had been approved, we just got on the internet and just, you know just looking around and that looked like a good programme.’

PwPD2

## 4 Discussion

This study was the first to explore the referral pattern of PwPD to allied health in Australia, by examining the journey of PwPD from diagnosis to the present. There was no consistent pattern to PwPD accessing allied health, with barriers and facilitators to access described using the Levesque model [56].

Many participants wished they had received more information about PD and allied health earlier in their journey. They recognised the importance of acceptance, maintaining a positive attitude [40], and engaging in exercise or physical activity [57], findings consistent internationally. Dissatisfaction with the education provided, particularly at diagnosis, and a desire for more resources have similarly been reported [39, 57].

The most frequently accessed allied health disciplines were PT, SP and OT [9, 11, 27], mirroring the traditional makeup of the MDT supported by substantial research evidence in PD management [10, 26, 58]. While some participants received an MDT assessment, the majority did not receive team‐based care, consistent with findings of low MDT referral rates internationally [11] and perhaps reflecting the lack of MDTs available for PwPD in Australia. Participants without access to private transport encountered significant difficulties accessing healthcare. This is common among PwPD worldwide [30], and particularly pronounced in regional Australia, where long travel distances further limit access.

There was no pattern to the timing of when participants accessed allied health, either physician or self‐referral, and not all participants were referred by other clinicians. Participants sought allied health both proactively and in response to symptoms. The most common trigger to seeking intervention was receipt of a funding package. The reactive nature of allied health services has been previously described [9, 25, 26, 31]. Participants were often unaware of the importance of receiving interventions from PD‐specialised clinicians, despite evidence showing experts provide higher‐quality care [26, 39].

All PwPD in this study demonstrated some ability to seek and engage with allied health, with a minority indicating limited health literacy. Reassuringly, PwPD sought information from reputable websites and PD support groups, similar to studies in the United Kingdom where most participants utilised the national PD website [57] and Malaysia where almost half of PwPD reported that support groups were their primary source of information [59]. In contrast, an international scoping review found that PwPD had unsatisfactory levels of health literacy, limited ability to seek and engage with general healthcare and that very few researched PD on their own [30]. In our study, 78% of PwPD had sufficient health literacy [48] and 89% had completed high school or higher education, indicating that our sample was likely more health literate than participants in the scoping review. A dependence of some PwPD on healthcare providers for education and referrals was a finding present both in our study and the review [30].

Affordability was a significant barrier to allied healthcare access for PwPD [59]. Participants described difficulties paying for care and insufficient private health insurance [30]. Receiving a funding package commonly triggered participants to seek care; however, the value of funding varied. PwPD using the NDIS accessed more allied healthcare than those using My Aged Care, likely due to the NDIS's ‘reasonable and necessary’ [60] funding model compared to capped allocations under My Aged Care. This disparity places PwPD aged ≥ 65 years at a disadvantage. However, even among NDIS recipients, funding was not always sufficient, leading to discontinued care. Therefore, there is a need to review and align funding models to ensure equitable access to allied healthcare across the lifetime of PwPD.

Supply‐side barriers for PwPD included a lack of availability of healthcare services, particularly in regional areas; long waitlists, especially for specialist care; and uncoordinated care, consistent with international experiences [30]. Some participants from CALD backgrounds expressed a preference for traditional medicine, similar to a Malaysian study of PwPD [59] where one‐third of participants considered such remedies, and in a study of general‐population South Asian migrants in Australia [61]. While this preference may be linked with health literacy, with more than half the participants in the Malaysian study expressing misconceptions about PD causes and a cure [59], health literacy among those who tried traditional medicine in our study was variable.

CALD PwPD in this study encountered language barriers when accessing healthcare, consistent with findings from Australian and European studies [62, 63]. Additional challenges, including navigating complex health systems [61, 62, 63], a preference for clinicians who speak their language [61] and reliance on social networks to assist in navigating healthcare access [61, 62], have also been reported. These language and cultural barriers compounded broader issues experienced by all participants, including the financial burden of healthcare [61, 62, 63], travelling long distances for access, particularly in regional areas [62, 63], and long wait times to access healthcare [61, 62].

### 4.1 Clinical Implications

Unlike neurological conditions with sudden onset, such as stroke or traumatic brain injury, where people are hospitalised and receive immediate, coordinated and ongoing allied healthcare [64, 65, 66], PwPD are typically diagnosed in the community and may not receive allied health until later stages. Consequently, there is a need for clinicians to provide PwPD education about allied health management at diagnosis and to direct them to reputable resources and support networks. As many PwPD are unaware of which allied health disciplines they will need, or the importance of PD‐specialised clinicians, proactive referral by other clinicians is essential. Referrals should occur both proactively from the time of diagnosis and in response to trigger events such as falls or swallowing problems.

Multidisciplinary care [26] is particularly important for people with young‐onset PD, as they will be living with a progressive disease for an extended time. Regular MDT follow‐up would ensure that potential problems are identified quickly and managed via intensive bouts of therapy [25, 26]. Geographically based MDTs throughout Australia, similar to the regional multidisciplinary networks of ParkinsonNet in the Netherlands [67], would enable PwPD to access appropriate local services and reduce approachability barriers. Ideally, PwPD should have their own case manager [26] who can assist them with navigating the complexities of the healthcare system and enable them to access MDTs.

The strengths of this study are that it included PwPD across the disease spectrum and with young‐onset PD. Additionally, the inclusion of CALD PwPD and their CPs broadens the findings of this study. Limitations include the fact that overall participants had high health literacy, education levels and socio‐economic status; no PwPD was in Hoehn and Yahr stage 5; and few CALD participants required interpreters. However, given that this highly educated and health‐literate group of participants, with high socio‐economic status, experienced significant barriers when accessing allied health, it is likely that barriers would be exacerbated in more disadvantaged PwPD.

## 5 Conclusion

This study highlighted that there is no structured, systematic process for referral of PwPD to allied health, resulting in PwPD accessing allied health reactively. PwPD encounter many barriers when attempting to access allied healthcare, as described by the Levesque model [56]. PwPD would prefer to receive information and referral to allied health earlier in their disease and should be able to access regular MDT care across their lifetime. A review of existing Australian funding arrangements should be undertaken to ensure they are better able to meet the needs of PwPD.

## Author Contributions

**Cassandra M. Wong:** conceptualization, formal analysis, funding acquisition, investigation, methodology, visualization, writing – original draft. **Sarah M. Dennis:** conceptualization, formal analysis, funding acquisition, supervision, methodology, writing – review and editing. **Natalie E. Allen:** conceptualization, formal analysis, funding acquisition, writing – review and editing, supervision. **Serene S. Paul:** conceptualization, formal analysis, funding acquisition, writing – review and editing, supervision.

## Ethics Statement

This study was approved by the University of Sydney Human Research Ethics Committee (2022/892). Access requests to distribute study information were approved for Westmead Hospital, Western Sydney Local Health District (ACCESS/24/WMEAD/5) and South Western Sydney Local Health District (AR24/001).

## Consent

All participants provided oral or written informed consent before data collection.

## Conflicts of Interest

The authors declare no conflicts of interest.

## Supporting information

Appendix A ‐ Materials and Methods.

Appendix B ‐ PD Diary.

Appendix C ‐ Interview Prompts.docx.

Appendix D ‐ Recruitment flowchart.

## Data Availability

The data that support the findings of this study are not publicly available due to ethical restrictions. De‐identified data is available upon reasonable request from the corresponding author.
