# Supplementary material for: ‘It Would've Been Nice to Know About Allied Health Earlier’: Insights From People With Parkinson's Disease
Source: Health Expect. 2025 Aug 21;28(4):e70391. doi: 10.1111/hex.70391 (PMC12368983; doi:10.1111/hex.70391)
Supplement: Supplementary file 1 — Appendix A ‐ Materials and Methods. [file HEX-28-e70391-s002.docx]

**Appendix A: Materials and Methods**

*Recruitment Methods*

Recruitment occurred via: advertisements distributed by Parkinson’s NSW and the Australian Physiotherapy Association, a database of PwPD willing to participate in research held by the researchers, and advertisements distributed by physiotherapists at sites within two Sydney Local Health Districts. Snowball sampling was also utilised, particularly for recruiting culturally and linguistically diverse (CALD) PwPD.

*Data Collection*

Level of physical activity was collected via the incidental and planned exercise questionnaire (IPEQ)^47^ for older people.

A single health literacy screening question rated participant’s confidence completing medical forms by themselves on a 5-point Likert scale ranging from “Not at all” to “Extremely”^48^. The “Somewhat” response has been identified as the best cut-off point for identifying limited or marginal health literacy skills^48^.

Disease severity was rated utilising the self-reported Hoehn and Yahr stages^45^.

Non-motor and motor experiences of daily living was collected using the Movement Disorders Society sponsored version of the Unified Parkinson’s Disease Rating Scale, Parts I and II (MDS-UPDRS-I and II)^49^.

Participants were sent the structured retrospective chart (“PD diary”) when they expressed interest in the study and the procedure was explained both verbally and in the Participant Information Statement so they were aware of the time commitment required to complete the chart. Participants who did not have the cognitive capacity to complete it alone could participate if a care-partner was willing to participate in the study with them. It was estimated that completing the chart would take between 10 and 30 minutes.

*CALD Participation in Study Procedures*

A phone or video interpreter was provided to CALD participants to explain the study, complete the consent forms, questionnaires, and interviews. The chart was available and completed in the participant’s preferred language. At the follow-up interviews (Interview-2), the participant read out the chart and the interviewer transcribed it into English with interpreter assistance.
