# Supplementary material for: ‘It Would've Been Nice to Know About Allied Health Earlier’: Insights From People With Parkinson's Disease
Source: Health Expect. 2025 Aug 21;28(4):e70391. doi: 10.1111/hex.70391 (PMC12368983; doi:10.1111/hex.70391)
Supplement: Supplementary file 2 — Appendix B ‐ PD Diary. [file HEX-28-e70391-s003.docx]

**INSTRUCTIONS**

1. Please fill in your name and indicate if someone helped you to complete this diary.
2. Each space represents 6 months as is written at the top of the table. Please fill in which year you are referring to at the top of the table.
3. Please indicate approximately which half of the year that events occurred, or that symptoms appeared or got worse. Do not worry if you have not yet experienced any of these symptoms. This is just a list to prompt your memory and it does not mean that you are going to develop these symptoms.
4. To help prompt your memory, you can chart important life events alongside which half of the year they occurred (eg, holidays, weddings, changing jobs). You may also want to refer to your own diary/diaries to help you complete this.
5. Please indicate if and when you fell, were hospitalised, had medication changes, required rehabilitation, got referred, saw allied health, etc.
6. A fall is defined as an event which results in you coming to rest inadvertently on the ground or floor or other lower level.

For example, *stumbling against the wall is not a fall.*

*Falling backwards unintentionally into a chair or lounge after standing up is a fall.*

*If you flop into the chair or lounge uncontrollably but you were intending to sit down, then that is not a fall.*

Please indicate when you started falling and when your fall frequency changed, ie, when you started falling more often such as weekly, or daily.

1. To indicate a change in symptoms, you can use arrows. Eg, ↑ for an increase and ↓ for a decrease.
2. If you need extra space to describe an event, you can use the notes section on the first page. You might like to write a (1) in the box, then write a (1) in the notes section so we know what point you are expanding on.
3. Please see below for an example year.

**Example Parkinson’s Disease Diary**

Name ___Jane Smith___ Did someone help you to complete this? (Please circle) Yes / No

|  | | **Year** 2021 | | **Year** 2022 | |
| --- | --- | --- | --- | --- | --- |
|  | | **Jan-Jun** | **Jul-Dec** | **Jan-Jun** | **Jul-Dec** |
| **Life Events** | |  | Grandson born |  | Holiday to Qld |
| **Health issues** | | Knee replacement (1) |  |  |  |
| **PD Medication Changes** | |  |  | Dose increased June (4) |  |
| **PD surgeries** | |  |  |  |  |
| **Falls** | |  |  |  |  |
| **First time I noticed**  **difficulty with, or when it changed (↑ or ↓)** | Speech |  |  |  |  |
|  | Saliva/drooling/swallowing |  |  |  |  |
|  | Showering/dressing |  |  | Trouble with buttons (3) | ↓ |
|  | Using my hand(s) |  | Noticed for the first time | ↑ Feb-May (3) | ↓ |
|  | Turning in/getting out of bed |  |  |  |  |
|  | Walking | (2) hard |  |  |  |
|  | Balance |  |  |  |  |
|  | Freezing |  |  |  |  |
|  | Community access | (2) hard |  |  |  |
|  | Other activities (eg, gardening, cooking, hobbies) Knitting  _________________  _________________ |  |  |  |  |
|  |  |  | Trouble making a jumper for the baby |  |  |
|  |  |  |  |  |  |
|  | Pain |  |  |  |  |
|  | Fatigue |  |  |  |  |
|  | Memory/thinking |  |  |  |  |
|  | Mood |  |  |  |  |
| I did not have difficulties with any activity | |  |  |  |  |
| **More / less physically active** | |  | Walked more (knee rehab) |  |  |
| **Allied Health** | | (1) for the knee |  |  | Occupational therapist (5) |

**Notes:** (1) In hospital for 5 days. Had physio and occupational therapy in hospital. Had physio appointments 2/week for a month after leaving hospital.

1. Both hard because of knee replacement. Was using a walker when I left the hospital and then a walking stick.
2. Tremor started in Feb, trouble with buttons in May.
3. Had follow-up with my Neurologist in June. Increased my medications and referred to occupational therapy.
4. Saw an occupational therapist for hand exercises.

|  | **Year_____________** | | **Year_____________** | |
| --- | --- | --- | --- | --- |
|  | **Jan-Jun** | **Jul-Dec** | **Jan-Jun** | **Jul-Dec** |
| **Life Events** |  |  |  |  |
| **Health issues** (Eg, other health conditions, major health issues, hospitalisation, other surgeries) |  |  |  |  |
| **PD Medication Changes** |  |  |  |  |
| **PD surgeries** (eg, DBS) |  |  |  |  |
| **Falls** |  |  |  |  |

**Notes:**

|  | | **Year_____________** | | **Year_____________** | |
| --- | --- | --- | --- | --- | --- |
|  | | **Jan-Jun** | **Jul-Dec** | **Jan-Jun** | **Jul-Dec** |
| **First time I noticed**  **difficulty with, or when it changed (↑ or ↓)** | Speech |  |  |  |  |
|  | Saliva/drooling/swallowing |  |  |  |  |
|  | Showering/dressing |  |  |  |  |
|  | Using my hand(s) |  |  |  |  |
|  | Turning in/getting out of bed |  |  |  |  |
|  | Walking |  |  |  |  |
|  | Balance |  |  |  |  |
|  | Freezing |  |  |  |  |
|  | Community access |  |  |  |  |
|  | Other activities (eg, gardening, cooking, hobbies)___________  _________________  _________________ |  |  |  |  |
|  |  |  |  |  |  |
|  |  |  |  |  |  |
|  | Pain |  |  |  |  |
|  | Fatigue |  |  |  |  |
|  | Memory/thinking |  |  |  |  |
|  | Mood |  |  |  |  |
| I did not have difficulties with any activity | |  |  |  |  |
| **More / less physically active** (eg, walking, exercise groups, use of personal trainer, dancing, yoga, gym, boxing etc) | |  |  |  |  |
| **Allied Health** (eg, physiotherapy, exercise physiology, occupational therapy, social work, speech pathology, dietetics, psychology) | |  |  |  |  |
