# Supplementary material for: ‘It Would've Been Nice to Know About Allied Health Earlier’: Insights From People With Parkinson's Disease
Source: Health Expect. 2025 Aug 21;28(4):e70391. doi: 10.1111/hex.70391 (PMC12368983; doi:10.1111/hex.70391)
Supplement: Supplementary file 3 — Appendix C ‐ Interview Prompts.docx. [file HEX-28-e70391-s004.docx]

**Appendix C: Interview Prompts**

The following prompts are indicative of what will be used during the semi-structured interviews:

**Interview-1**

- What did your healthcare provider tell you about the disease when you received your diagnosis? And who was the healthcare provider that provided this information?
- Was your healthcare provider keen to bring in other healthcare services? And when?
- How did you seek information on PD and learn about its management? What have you learnt about the management of PD?
- Who is the main person who manages your PD? Is a PD nurse involved in your care?
- When did you realise you needed other health care professional involvement? Is this when you were referred? This question may alternatively be asked after the diary is introduced, or in the second interview session
- If you have not had other health care professional involvement, have you felt as though you have needed it? This question may alternatively be asked after the diary is introduced, or in the second interview session
- Was there anything in particular that triggered a need for allied health involvement/exercise etc? Prompt for specific disciplines. This question may alternatively be asked after the diary is introduced, or in the second interview session
- Have you seen any complementary/alternative therapists (eg, acupuncture, osteopath, faith healer)?
- Medication-related questions – at this stage, the diary may be introduced and used as a prompt
  - When did medication usage start to increase?
  - Did an increase in medication usage coincide with when you thought you needed, or when you were referred to allied health?

**Interview-2**

- When did you realise you needed other health care professional involvement? Is this when you were referred?
- If you have not had any other health care professional involvement, have you felt as though you have needed it? Why have they not been involved?
- Was there anything in particular that triggered a need for allied health involvement/exercise etc?
- Have you ever received contradictory information from the health professionals involved in your care?
- Do you feel that the care you receive from the different health professionals managing your PD is continuous/seamless, or fragmented?

Additional participant specific prompts will be tailored based off the information provided in the PD diary (eg, hospitalisations, worsening of symptoms).
