# Supplementary figures and images for: ‘It Would've Been Nice to Know About Allied Health Earlier’: Insights From People With Parkinson's Disease
Source: Health Expect. 2025 Aug 21;28(4):e70391. doi: 10.1111/hex.70391 (PMC12368983; doi:10.1111/hex.70391)

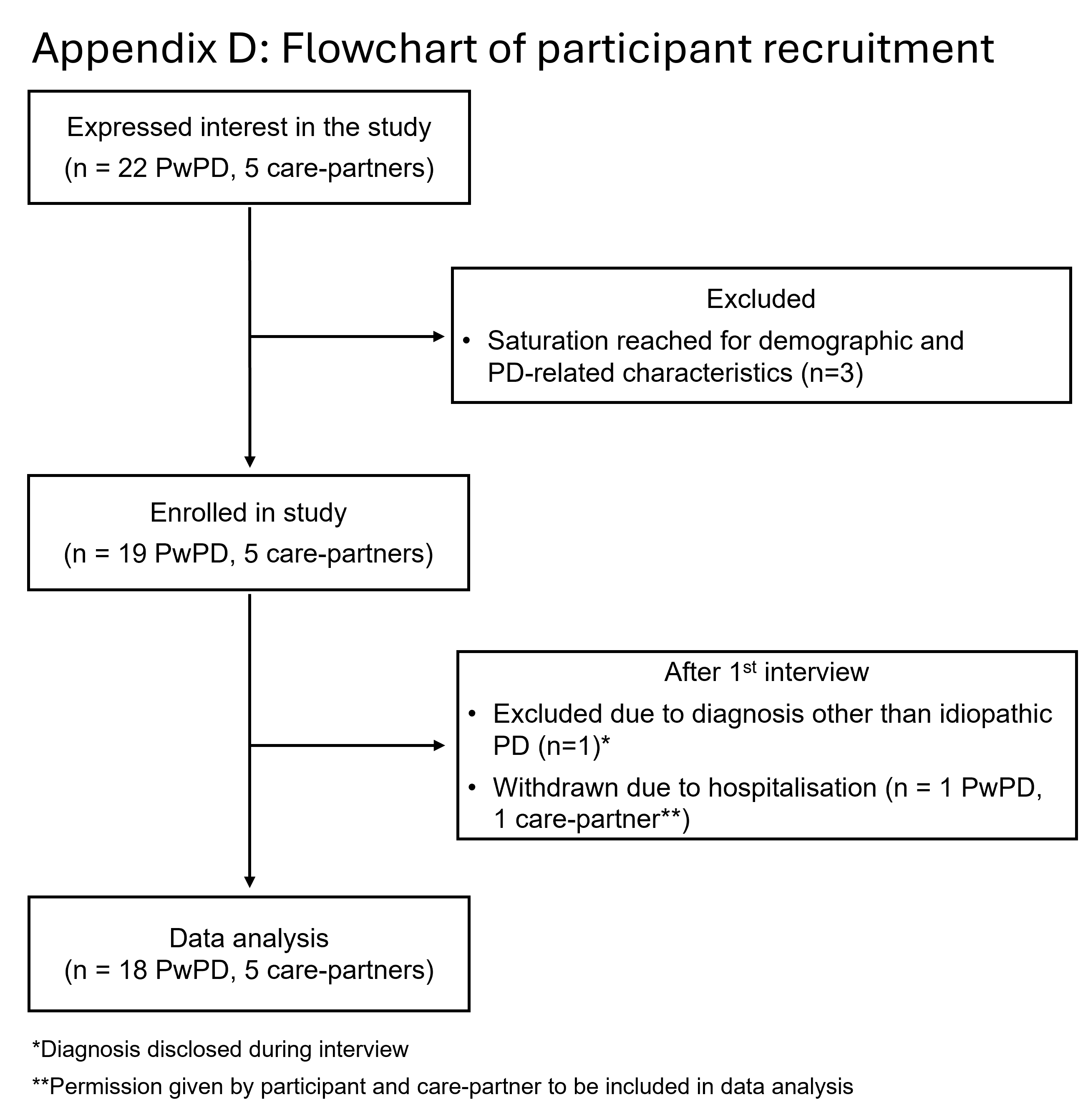

Supplement: Supplementary file 4 — Appendix D ‐ Recruitment flowchart. [file HEX-28-e70391-s001.png]
